# The Interplay between Natural Selection and Susceptibility to Melanoma on Allele 374F of *SLC45A2* Gene in a South European Population

**DOI:** 10.1371/journal.pone.0104367

**Published:** 2014-08-05

**Authors:** Saioa López, Óscar García, Iñaki Yurrebaso, Carlos Flores, Marialbert Acosta-Herrera, Hua Chen, Jesús Gardeazabal, Jesús María Careaga, María Dolores Boyano, Ana Sánchez, Juan Antonio Ratón-Nieto, Arrate Sevilla, Isabel Smith-Zubiaga, Alicia García de Galdeano, Conrado Martinez-Cadenas, Neskuts Izagirre, Concepción de la Rúa, Santos Alonso

**Affiliations:** 1 Department of Genetics, Physical Anthropology and Animal Physiology, University of the Basque Country UPV/EHU, Leioa, Bizkaia, Spain; 2 Ertzaintza Forensic Unit, Erandio, Bizkaia, Spain; 3 CIBER de Enfermedades Respiratorias, Instituto de Salud Carlos III, Madrid, Spain; 4 Research Unit, Hospital Universitario N.S. de Candelaria, Tenerife, Spain; 5 Applied Genomics Group (G2A), Genetics Laboratory, Instituto Universitario de Enfermedades Tropicales y Salud Pública de Canarias, Universidad de La Laguna, Tenerife, Spain; 6 Research Unit, Universitary Hospital Dr. Negrin, Las Palmas de Gran Canaria, Spain; 7 Center for Computational Genetics and Genomics, Temple University, Philadelphia, Pennsylvania, United States of America; 8 Dermatology Service, BioCruces Health Research Institute, Cruces University Hospital, Cruces-Barakaldo, Bizkaia, Spain; 9 Dermatology Service, BioCruces Health Research Institute, Basurto University Hospital, Bilbao, Bizkaia, Spain; 10 Department of Cell Biology and Histology, University of the Basque Country UPV/EHU, Leioa, Bizkaia, Spain; 11 Department of Zoology and Animal Cell Biology, University of the Basque Country UPV/EHU, Leioa, Bizkaia, Spain; 12 Department of Medicine, Jaume I University of Castellón, Castellón, Spain; University of Lausanne, Switzerland

## Abstract

We aimed to study the selective pressures interacting on *SLC45A2* to investigate the interplay between selection and susceptibility to disease. Thus, we enrolled 500 volunteers from a geographically limited population (Basques from the North of Spain) and by resequencing the whole coding region and intron 5 of the 34 most and the 34 least pigmented individuals according to the reflectance distribution, we observed that the polymorphism Leu374Phe (L374F, rs16891982) was statistically associated with skin color variability within this sample. In particular, allele 374F was significantly more frequent among the individuals with lighter skin. Further genotyping an independent set of 558 individuals of a geographically wider population with known ancestry in the Spanish population also revealed that the frequency of L374F was significantly correlated with the incident UV radiation intensity. Selection tests suggest that allele 374F is being positively selected in South Europeans, thus indicating that depigmentation is an adaptive process. Interestingly, by genotyping 119 melanoma samples, we show that this variant is also associated with an increased susceptibility to melanoma in our populations. The ultimate driving force for this adaptation is unknown, but it is compatible with the vitamin D hypothesis. This shows that molecular evolution analysis can be used as a useful technology to predict phenotypic and biomedical consequences in humans.

## Introduction

Adaptation to new environments is key to species survival. The adaptive nature of pigmentation in humans was already suggested by Relethford [1], who observed that 88% of total variation in skin color is due to differences among major geographic groups, contrary to other neutral genetic markers and DNA polymorphisms which show most of their diversity, instead, within local populations. The adaptive nature of skin pigmentation is twofold. On the one hand, it has been proposed that early humans living in Africa had a pigmented skin that conferred protection against the damaging effects of ultraviolet (UV) radiation, including sunburns [2], skin cancer [3] and/or the photolysis of folate, an essential vitamin to fetal development and male fertility [4]. On the other hand, it has also been long assumed that the settlement of human populations in regions of higher latitudes, where the intensity of incident UV radiation was lower, brought along the depigmentation of the human skin. However, in such scenario, it still remains a source of debate whether the depigmentation process would reflect a relaxation of functional constraints, or if it indeed conferred a selective advantage, presumably as a mechanism to enable the synthesis of the appropriate levels of vitamin D [4],[5],[6].

Although there are over 100 genes related to the pigmentary phenotype in mice [7], only a handful have been shown so far to have effects on normal variation in pigmentation in humans (See [8] for a review of pigmentation-associated mutations in humans, mice and other mammals). The strongest evidences are found in the pigmentary genes *MC1R* [9],[10], *ASIP* [11],[12], *SLC24A5* [13], *SLC45A2* [14], *TYR* [15],[16],[17], *OCA2* [18],[19],[20] and *KITLG* [21]. Among these, *SLC45A2* has a major function in the process of melanin synthesis by controlling the activity and traffic of tyrosinase to the melanosomes, and maintaining the melanosomal pH [14], [22], [23]. *SLC45A2*, also known as *MATP or AIM1*, is a membrane associated transporter gene located on chromosome 5p and consists of seven exons spanning a region of approximately 40 kb. Mutations in this gene can cause type 4 oculocutaneous albinism (OCA4) in humans [24] and other primates [25].

Graf et al. [14] first revealed an association of two common single nucleotide polymorphisms (SNPs) in *SLC45A2*, Leu374Phe (L374F, rs16891982) and Glu272Lys (E272K, rs26722), with human pigmentation variation in European descents from Australia (presumably of North European origin). It has been proposed that the ancestral 374L allele, which is fixed in African populations, would contribute to an optimal eumelanin production, while the 374F allele, which is almost fixed in European populations, would originate an acidic melanosomal environment that negatively affects tyrosinase activity, hence leading to a lighter pigmentation [23]. Lucotte et al. [22] showed a broad-scale latitudinal gradient for the frequencies of the 374F allele, from the Northern Africa to Europe, thus reinforcing the role of this variant in the depigmentation process of Europeans. In the same vein, Soejima et al. [26] showed evidences of positive selection acting on this gene in a sample of European-Africans. Prompted by these observations, we aimed to perform an integrative and exhaustive analysis of the selective pressures acting on specific variants of *SLC45A2* in a South European population.

Furthermore, the involvement of genetic variants of *SLC45A2* in melanoma susceptibility is also being investigated. In fact, the variant 374L has been shown to be protective against melanoma in different European populations [27],[28],[29]. We have recently shown the presence of signatures of positive selection acting over the pigmentation and melanoma-risk locus *MC1R* in Europeans [30]. Motivated by this interplay between selection and susceptibility to disease, here we aimed to provide full comprehension of how the interaction between natural selection and disease susceptibility has shaped the genetic variation of *SLC45A2* in a South European population (Spain) at intermediate latitude between Northern Europe and Africa.

## Results

### Population structure analysis

From a total of 500 Spanish individuals sampled, we selected the 34 most and 34 least pigmented individuals (below percentile 31 and above percentile 83 of the distribution of reflectance values, respectively) to analyze the association of *SLC45A2* to skin pigmentation. Before that, we performed a series of tests to verify the absence of population structure, therefore preventing false positive results. Thus, these 68 individuals were genotyped using the Genome-wide Human SNP Array 6.0 (Affymetrix), and after data management with PLINK, a total of 106,521 SNPs were considered for stratification analysis using the twstats program implemented in EIGENSOFT. No significant principal component was identified (Tracy-Widom test, p-value = 0.418 for the first PC; p-value>0.998 for the remaining PCs) suggesting a lack of population stratification. Furthermore, the QQ-plot generated with the p values for each SNP (Figure S1) showed an overlap between the expected and observed p values, indicative of lack of stratification, and supported by the value of the inflation factor which was of 1.

In order to confirm these results, we performed a third analysis based on STRUCTURE. For that, we additionally genotyped 15 STRs in the above samples. No significant deviations from Hardy–Weinberg expectations based on the Exact Test were found in the STRs analyzed. As the power to detect population structure is highly dependent on the number of loci utilized we also resampled different subsets of SNPs (200, 500 and 1,000) previously genotyped with the Genome-wide Human SNP Array 6.0 (Affymetrix). In all the cases (STRs and SNPs), and using correlated allele frequencies and an admixture model, log-likelihood scores suggested that the global maximum was reached when the number of assumed populations (k) was equal to 1. This was consistent with the hypothesis that the sample under study was genetically homogeneous.

**Figure 1:**
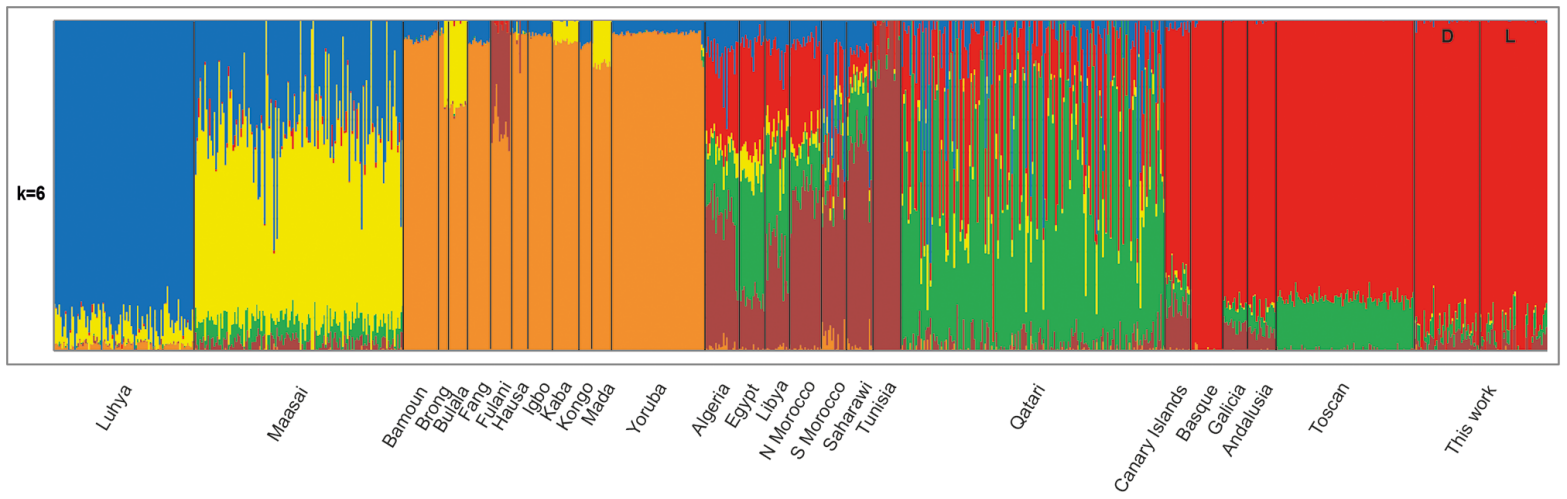
Admixture map for ancestral populations (k) = 6. Each vertical line represents an individual from the corresponding population. Different colors indicate the ancestry proportions. The samples inside the black square correspond to the samples analyzed in this work. D: most pigmented individuals from our samples, L: least pigmented individuals from our sample.

The absence of genetic structuration was also confirmed by a fourth analysis that used ADMIXTURE. In this case, to improve ancestry assignments we also included samples from different world populations genotyped with the same array (see Materials and Methods and Table S1 for more details). 10 ancestral populations (k = 2–10) were tested, performing 10 iterations with random seeds for each k value. As seen in Figure S2, cross validation error for ADMIXTURE was the lowest at k = 6, showing a subtle difference with k = 7. Population structure, as inferred by the analysis at k = 6, is shown in Figure 1. The results of the analysis at k = 7 are shown in Figure S3. Again, we observed that the most and the least pigmented individuals in our sample were homogeneous, sharing a predominant European genetic component without significant African contributions.

### Resequencing of the coding region of *SLC45A2* in Spanish samples

**Figure 2:**
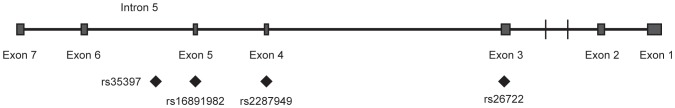
SNPs found in the resequencing of *SLC45A2*. We show the location of the 3 SNPs found in the coding region of the gene: rs16891982, rs2287949 and rs26722, plus the most frequent SNP in intron 5: rs35397.

Having proved the homogeneity of the sample, we proceeded to resequence the complete coding region and the 5′UTR of *SLC45A2* in the selected 34 most and 34 least pigmented individuals. Resequencing revealed only 3 exonic SNPs (Figure 2), which had already been reported elsewhere: E272K (rs26722), T329T (rs2287949) and L374F (rs16891982), with a frequency of the derived alleles in our sample of 68 individuals of 0.052, 0.022 and 0.882, respectively. As T329T is a synonymous mutation and it has a low frequency, and E272K has also a low frequency, only the L374F polymorphism was considered for further analysis. The 374F variant of this SNP showed statistically significant differences between the most and the least pigmented individuals (Fisher exact test, p = 0.001). In the group of the most pigmented individuals (reflectance ranging from 60.67 to 68.43, measured at 685 nm), the variant 374F appeared at a frequency of 0.794, while in the group of the least pigmented individuals (reflectance from 74 to 79.67, at 685 nm), the frequency of 374F was 0.971 (Table 1). Haplotype frequency differences among the groups of most and least pigmented individuals are shown in Table S2.

**Table 1:** Frequency of 374F (rs16891982) and the A allele (intronic rs35397) in a) the most pigmented (Dark) and least pigmented (Light) individuals of the skin reflectance distribution from our study and in b) the populations from 1KGP.

	rs16891982 (L374F)			rs35397 (C/A)		
Samples	n	374F	p	n	A	p
**a) Reflectance**						
Dark (60.67–68.43)	68	0.7941	0.0001	68	0.75	<0.0001
Light (74–79.67)	68	0.9705		68	0.96	
**b) 1KGP**						
Africa	492	0.059		492	0.096	
Europe	760	0.971		760	0.949	
- North Europe^1^	536	0.976		536	0.948	
- South Europe^2^	224	0.96		224	0.951	
East Asia	570	0.017		570	0.028	

n: number of chromosomes.

1 North Europe: CEU (Utah residents with Northern and Western European Ancestry, n = 174), GBR (British in England and Scotland, n = 176) and FIN (Finnish in Finland, n = 186).

2 South Europe: TSI (Toscani in Italy, n = 196) and IBS (Iberian in Spain, n = 28).

In order to compare our results with other populations worldwide, we obtained the allele frequencies for this SNP in Africans, Europeans and East Asians from 1KGP (1000 Genomes Project) (Table 1). The frequency for the variant 374F in the group of the lighter individuals was similar to the frequency found in Europeans. The frequency of 374F in the group of the darker individuals, however, differed notably from the 1KGP European sample.

### Association of L374F with hair and eye color

In order to assess also the association of L374F to hair or eye color, we genotyped a subset of 344 individuals from which we had paired information for these traits. We observed that the ancestral allele G (374L) was associated with black (OR = 2.14; p = 0.0018) and dark brown hair (OR = 2.24; p = 0.0189), and the darkest eye color (brown/black; OR = 1.89; p = 0.0082) (Table S3).

### Geographical distribution

**Figure 3:**
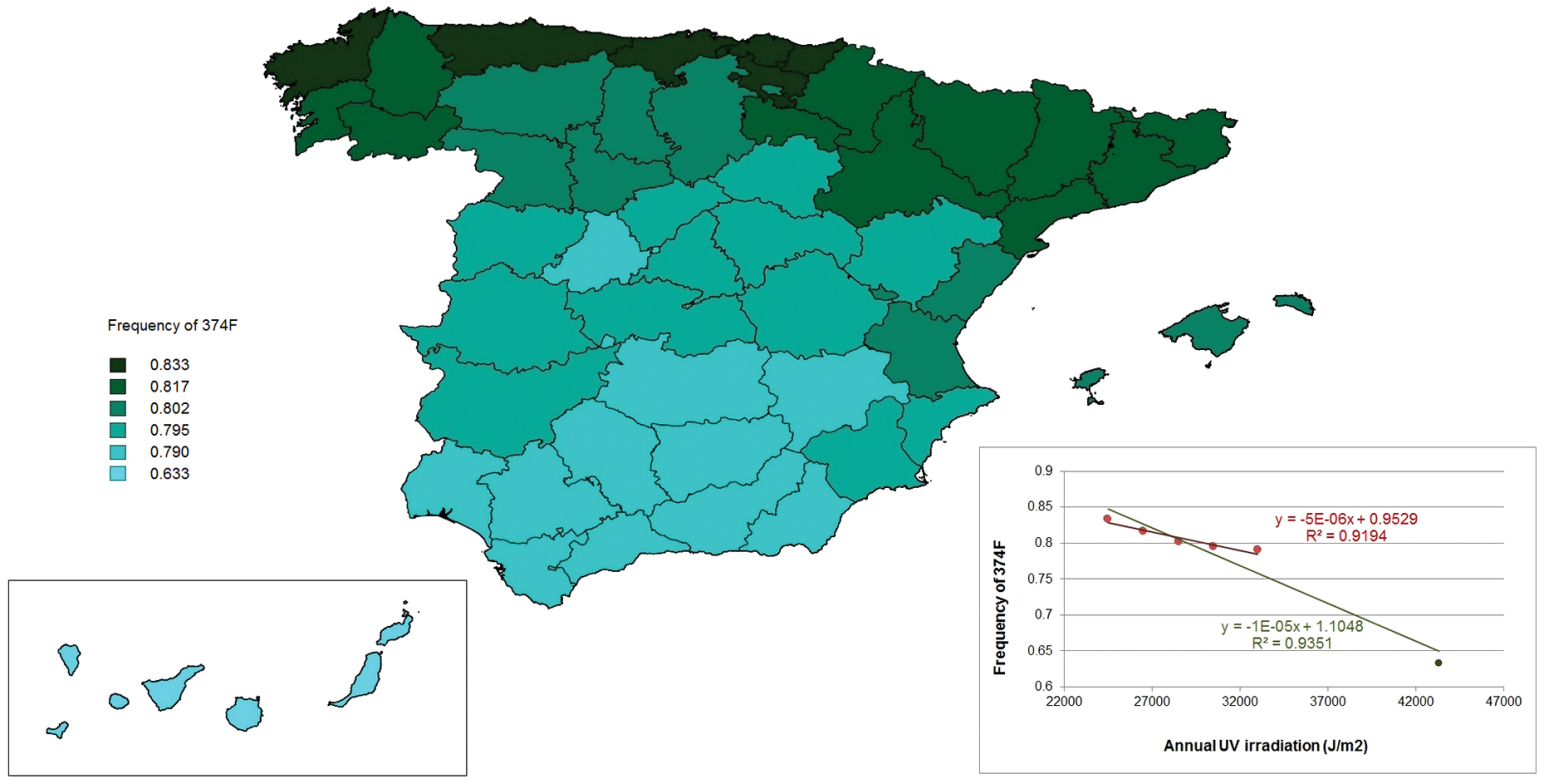
Frequency of 374F in Spain according to the intensity of annual UV irradiation. A colored map showing the frequency of 374F per UV intensity range and the correlation equations between UV intensity and frequency of 374F. UV ranges (J/m^2^) include: 23500–25500; 25501–27500; 27501–29500; 29501–31500; 31501–34500; and 43300. The green line corresponds to the correlation equation obtained when the Canary Islands were included. The red line corresponds to the correlation equation after excluding the Canary Islands.

Next, we wanted to assess if the frequencies of L374F correlated to incident UV radiation, over the Spanish geography. We genotyped a total of 528 individuals from different regions of the Iberian Peninsula with at least two generations of ancestry in their province of origin plus 30 individuals from the Canary Islands. We calculated the frequency of allele 374F for each province in the Iberian Peninsula (45 provinces in total) and because sample size per province was small, we grouped them according to homogeneous groups of UV radiation, which ranged from 23,500 to 34,500 J/m^2^. Annual UV irradiation in the Canary Islands was 43,400 J/m^2^. We obtained a decreasing gradient of 374F frequencies from the North to the South of Spain (Figure 3). We calculated the correlation coefficient (*r*) in two different scenarios: a) excluding the Canary Islands and b) including the Canary Islands. In both cases *r* was higher than 0.95 and significant (p<0.005) (Figure 3). UV irradiation is strongly related with latitude, which could lead to thinking that a demographic process is actually driving the distribution of frequencies in Spain. However, we have demonstrated in a previous paper [30] by means of principal component analyses based on data derived from 93 European ancestry informative markers that there is no stratification in the Spanish population. Therefore, the frequency of L374F in Spain does not correlate to latitudinal demographic processes that might have shaped the distribution of frequencies. Only an environmental variable can explain this correlation, being UV incidence the most likely candidate.

### Resequencing of the intron 5 of *SLC45A2*

Due to the low diversity found in the exonic regions, intron 5 of *SLC45A2* (4,180 bp) was also resequenced in the same 68 individuals (34 most and 34 least pigmented). We chose this intron as it was close to SNP L374F, and it was long enough to find sufficient variability to perform subsequent selection tests. All SNPs found in the intron 5, a total of 10, had been previously reported in dbSNPs: rs250416, rs142167897, rs35394, rs35395, rs142639084, rs35396, rs10080040, rs40132, rs35397 and rs115658239. Among these, the most polymorphic SNP was rs35397 (C/A) (Figure 2). The frequencies of the other non-significant SNPs are shown in Table S4. In this SNP, the derived allele (A) was almost fixed in the group of the light skinned pigmented individuals (p(A) = 0.96), while the frequency of this allele was significantly lower in the darker individuals (p(A) = 0.75) (Fisher's Exact test p<0.0001) (Table 1). The extent of linkage disequilibrium between the intronic SNP rs35397 and the coding SNP L374F in exon 5 (separated by 577 bp) was assessed in the sample of 68 individuals, showing a high D' value of 0.77 and a moderate *r* ^2^ value of 0.454. Haplotype frequency differences among the groups of most and least pigmented individuals are shown in Table S2. We also compared the frequency of this SNP in our sample with the populations from 1KGP and we observed that, similarly to SNP L374F, the frequency in the fair skinned individuals was similar to that in the European sample from 1KGP (0.960 vs. 0.949, respectively; Z-test p-value: 0.690), while the frequency in the most pigmented individuals was significantly lower (0.750 vs 0.949; Z-test p-value <0.0001) (Table 1). The derived allele is almost absent in the 1KGP African and Asian populations, thus suggesting a putative mechanism of depigmentation specific of Europeans.

### Sequence diversity and selection tests

The coding region of *SLC45A2* showed a low diversity in both the sample from Spain and the 1KGP European samples (Table 2). Due to this lack of diversity, neutrality tests (Tajima's D, Fu & Li's D and Fay & Wu's H) failed to detect any signature of selection acting on this gene based on coding regions. Intron 5, however, showed a high diversity in the Spanish and European samples from 1KGP. After Bonferroni correction, Tajima's D test was significant for the least pigmented (light) Spanish individuals and Europeans from 1KGP (both South and North) (Table 2). Fu & Li's D test was also significant for the group of least pigmented individuals from Spain. Although Tajima's D is more powerful to detect selective sweeps than other tests, it can also be confounded by demographic processes [31]. Therefore, we used the DH software to calculate the p-values of different selection tests under a model that incorporates a recent Out-of-Africa demographic history [32]. The p-value for the combined DHEW test [33], that is specific of recent positive selection, was significant for all the groups (see Table 2). However, after a Bonferroni correction for multiple tests, only the least pigmented individuals from Spain and South Europeans remained significant. Given that this intron is flanking the exon containing SNP L314F, which has been nearly fixed in Europeans and with a functional importance in the process of depigmentation, it is very likely that the evidences of positive selection found in intron 5 are due to the effect of selection on L314F.

**Table 2:** Diversity parameters and selection test for the coding region and intron 5 of *SLC45A2* gene.

Coding region							
Population	n	SS	pi	Hd	TD (p)	FLD (p)	FWHn (p)
**Spain (All)**	136	3	0.00236	0.320	−0.609 (n.s.)	−0.086 (n.s.)	0.049 (n.s.)
**Spain (Dark)**	68	3	0.00035	0.462	−0.303 (n.s.)	−0.079 (n.s.)	0.037 (n.s.)
**Spain (Light)**	68	3	0.0001	0.142	−1.484 (n.s.)	−0.031 (n.s.)	0.062 (n.s.)
**EU 1KGP (N+S)**	760	3	0.00005	0.079	−1.078 (n.s.)	−0.024 (n.s.)	0.019 (n.s.)
**EU 1KGP (N)**	536	3	0.00004	0.069	−1.184 (n.s.)	−0.028 (n.s.)	0.028 (n.s.)
**EU 1KGP (S)**	224	3	0.00007	0.069	−1.220 (n.s.)	−0.001 (n.s.)	0.042 (n.s.)

Intron 5								
Population	n	SS	pi	Hd	TD (p)	FLD (p)	FWHn (p)	DHEW p
**Spain (All)**	136	10	0.00017	0.294	−1.546 (p: 0.027)	−0.04 (n.s.)	−1.749 (0.051)	0.023
**Spain (Dark)**	68	9	0.00025	0.447	−1.197 (p: 0.092)	−0.89 (n.s.)	−1.698 (0.072)	0.040
**Spain (Light)**	68	8	0.00006	0.115	−2.173 (**p: 0.0004** *)	−2.04 (**0.004** *)	−1.921 (0.016)	**0.00025** *
**EU 1KGP (N+S)**	760	17	0.00008	0.179	−1.983 (**p: 0.002** *)	0.00442 (n.s.)	−1.830 (0.052)	0.019
**EU 1KGP (N)**	536	15	0.00008	0.187	−1.980 (**p: 0.0** *)	−0.01711 (n.s.)	−1.958 (0.023)	0.017
**EU 1KGP (S)**	224	11	0.00009	0.162	−1.884 (**p: 0.007** *)	−0.03072 (n.s.)	−2.221 (0.034)	**0.0009** *

*significant after Bonferroni correction.

**n.s: not significant.**

**n**: number of chromosomes; **SS**: segregating sites; pi: nucleotide diversity; **Hd**: haplotype diversity; **TD (p)**: Tajima's D (p value); **FLD (p)**: Fu & Li's D (p value); **FWHn (p)**: normalized Fay & Wu's H (p value); **DHEW(p)**: combined test DHEW (p value).

(p values in dnaSP obtained from 5000 standard coalescent simulations).

### Positive selection acting on 314F in South Europeans

**Figure 4:**
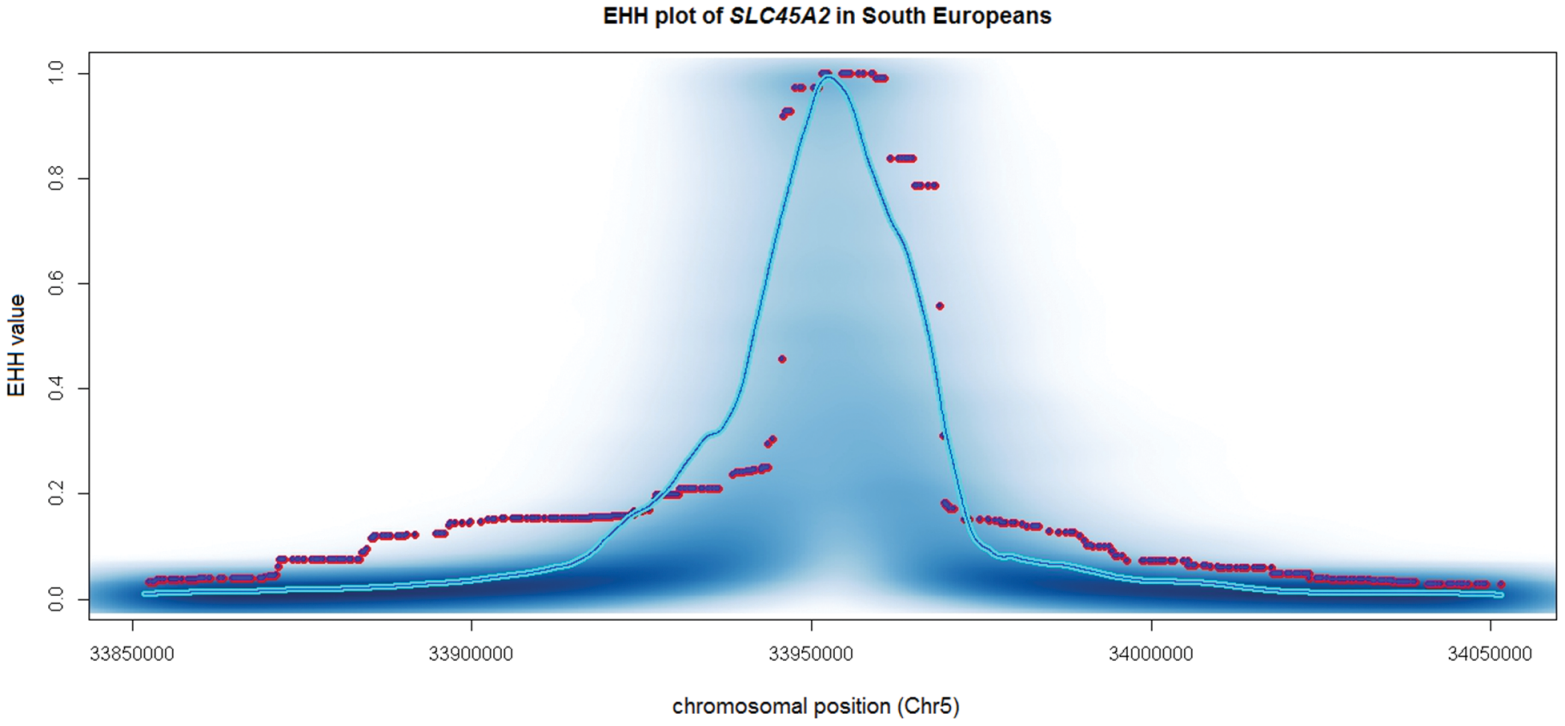
EHH analysis of the 1KGP South European samples. Simulated EHH values for the 374F haplotype using the demographic model of Gutenkunst et al. (2009). Shades of blue correspond to a smoothed color density representation of the scatter plot of all EHH simulations for each region. Blue darkness is proportional to the density of points in that vicinity. The continuous blue line corresponds to the 95th percentile of the distribution. Purple dots correspond to the observed values from the 1KGP data for South Europeans.

We investigated if recent positive selection could be acting specifically on the 374F variant by applying the EHH test to the Southern European sample (TSI and IBS) from 1KGP. Figure 4 shows that the extent of homozygosity from L374F is, in fact, longer than that expected under the neutral demographic model considered. This also shows that the variant 374F has been subject to recent positive selection.

To confirm this, we further analyzed a region of 5 kb upstream L314F in the South European populations from 1KGP and performed a haplogroup-specific Tajima's D test. Tajima's D test for the ancestral L314 haplogroup was 0.687 (p = 0.736), whereas for the derived haplogroup 314F it was −1.718 (p = 0.03). The negative and significant value of the Tajima's D test in the haplogroup 314F reinforces the idea that positive selection is acting on the derived allele of rs16891982 (L314F) in South Europeans.

### Estimation of the age of expansion of allele 374F

**Figure 5:**
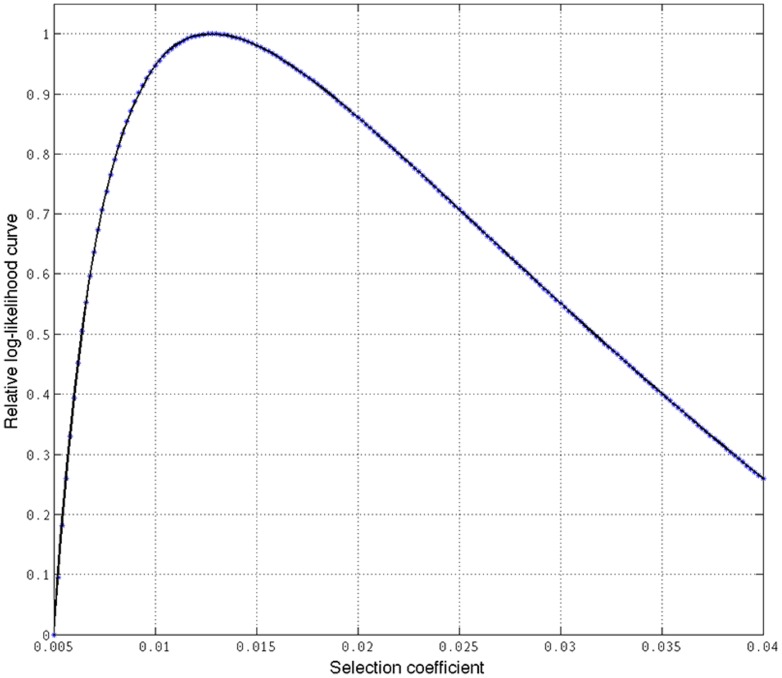
Estimation of selection coefficient (s) of allele 314F using the haplotipic data of 82 unrelated CEU individuals from the 1KGP. The highest likelihood was obtained for s = 0.0127 (95% CI, 0.0106–0.0148).

**Figure 6:**
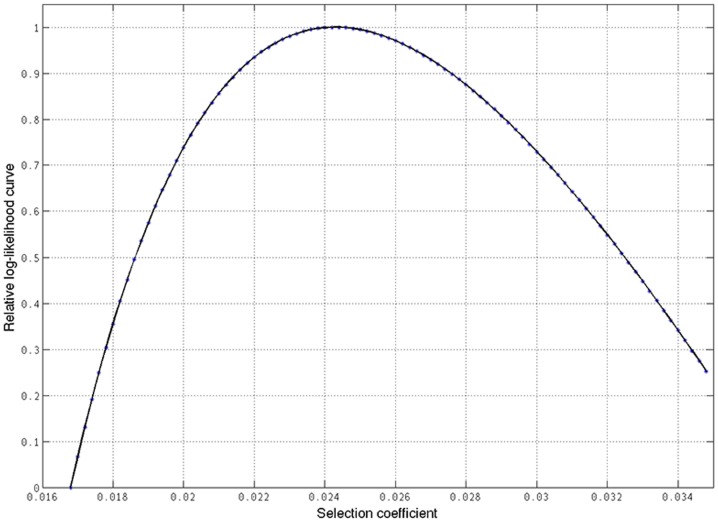
Estimation of selection coefficient (s) of allele 314F using the haplotipic data of 60 unrelated CEU individuals from the HapMap. The highest likelihood was obtained for s = 0.0243 (95% CI, 0.0111–0.0375).

To assess the meaning of 374F within the evolutionary history of Europeans we decided to estimate the selection coefficient and the age of expansion of this allele. We used 2 datasets: individuals from the 1KGP and HapMap databases (see Materials and Methods). Using the 1KGP data, the highest likelihood for the estimation of the selection coefficient was of 0.0127 (95% CI, 0.0106–0.0148) (Figure 5). The age of the expansion of the allele, assuming a generation time of 29 years was estimated to be of 29,450 years (95% CI, 25,270–35,290). Using the HapMap data, the highest likelihood for the estimation of the selection coefficient was of 0.0243 (95% CI, 0.0111–0.0375) (Figure 6). The age of the expansion of the allele in this case was estimated to be of 16,480 years (95% CI, 10,680–36,070). Thus, even with the uncertainty in the ranges provided by the two different datasets, due to the differences in the estimation of the coefficient of selection, this places the date for the onset of selective sweep of allele 374F in Europe in a period after 36,000 years ago, after the Out of Africa event.

### Association with melanoma

Given the association of this polymorphism (L374F) with pigmentation we wanted to assess next if L374F could be also associated to risk of melanoma in the Spanish population. For that, 119 melanoma samples were also genotyped for SNP rs16891982. We used as healthy controls all the samples analyzed in this work (the 68 individuals resequenced and the 344 individuals genotyped from our sample plus the 528 individuals from the Spanish National DNA Bank plus the 30 individuals from the Canary Islands). The genotypes obtained for each group are shown in Table 3. There was no evidence of departure from Hardy Weinberg Equilibrium in any of the groups. Interestingly, the homozygous genotype for the 374L allele was absent in all the melanoma samples. We found the L374F SNP to be significantly associated with melanoma, with the 374F (the “light” pigmentation allele) constituting a risk factor for melanoma (Cochran-Armitage Trend Test assuming an additive model, p-value: 4.36E-06). As we had observed non-uniform frequencies of this allele across the geography according to the intensity of the incident UV radiation, we decided to perform the association test considering different control groups: the populations with the highest and those with the lowest incidence of UV irradiation. Cochran-Armitage Trend Test was significant in all cases after Bonferroni correction (Table 3), supporting a strong significant association with melanoma after accounting for population differences in allele frequencies for this locus.

**Table 3:** Genotypic frequencies for melanoma samples and controls.

	374F/374F	374F/374L	374L/374L	HW(p)	C-A(p)
Melanoma	107	12	0	1	
Controls					
All samples	659	276	35	0.412	**1.42E-06** *
UV: 23500–25000 J/m^2^	43	14	3	0.190	**7.63E-04** *
UV: 31501–34500 J/m^2^	81	34	9	0.059	**9.11E-07** *
UV: 43387 J/m^2^	12	14	4	1	**4.86E-09** *

*significant after Bonferroni correction.

**HW(p)** = Hardy-Weinberg Exact Test (p value); **C-A(p)**: Cochran-Armitage test (p value).

## Discussion

Several studies have reported polymorphisms in *SLC45A2* with significant allele frequency differences between major human populations [34],[35]. Graf et al. [14] were the first to reveal an association between *SLC45A2* alleles and intrapopulation pigmentation variation based on E272K and L374F allele frequency differences, showing that alleles 374L and 272K were significantly associated with dark hair, skin, and eye color in North Europeans. In this study, we have analyzed the involvement of genetic polymorphisms in *SLC45A2* in the variability of human skin pigmentation in Spain, a South European population. By DNA resequencing of the most and least pigmented subsets of individuals in our sample, we showed that the 374L allele was significantly associated with darker skin color also in Southern Europe. The E272K polymorphism was also detected in our samples but at a low frequency; and it was not associated with pigmentation variation as reported by Graf et al. [13]. This is in agreement with more recent studies that indicated a lack of correlation between this variant and pigmentation [36],[37].

In relation to the role of L374F in pigmentary phenotypes, the results reported so far for its associations are slightly contradictory. In this study, we observed that the 374L allele was associated with dark skin, eye and hair color. Our results agree with those reported by Graf et al. [14], who found a strong association of these phenotypes with individuals of North European ancestry. However, other authors also supported its association with hair and skin pigmentation, but not with eye color [38]. On the other hand, Branicki et al. [36], found a strong association between 374L and dark hair color, but their results did not reveal an association with skin pigmentation. Some of the reasons that were proposed for this discrepancy included the low frequency of the 374L variant in their population, suggesting that hidden population stratification might be present in the study or, alternatively, that hair and skin color must be under the control of different genes and/or the presence of allelic heterogeneity [36].

A broad-scale latitudinal gradient for the frequencies of the 374F allele, from the Northern Africa to Europe has been previously described. Lucotte et al. [22] studied the distribution of the 374F allele in a total of 2063 individuals from 21 populations ranging from Northwestern Europe to North Africa, including 130 subjects from Spain (71 individuals from Sevilla and 59 from Barcelona) and found a remarkable decreasing gradient of this variant associated to the geographical latitude across the continent. The allele 374F was found to be nearly fixed in Northern European populations with decreasing frequencies towards the South. In addition, this variant has shown to be almost absent in Asian, Native American or African populations [14],[35],[39],[40], [41],[26],[22]. Thus, we wanted to assess the existence of this gradient in our Spanish population. Instead of the latitude, we decided to study the correlation of the frequency of 374F with the annual incidence of UV in each region, which may shed more light to its evolutionary meaning. Interestingly, we also obtained a significant correlation between the annual UV incidence and the frequency of 374F in Spain, where a decreasing pattern of 374F frequency with UV can be noted. To our knowledge, this is the first time that a correlation between the allele frequency and variation in UV intensity in such a small geographical region has been reported.

Soejima et al. [26] suggested that positive selection has been recently acting or has acted on this region of *SLC45A2*, and that the advantageous haplotype would have spread rapidly in Europe. They sequenced the exonic regions of *SLC45A2*, and also introns 3 to 5 and part of intron 6, and calculated nucleotide diversities for a chosen region of 7,551 bp surrounding L374F polymorphism, in order to find specific patterns of variation in the different populations analyzed. Their results for this region revealed deviations from neutrality in Europeans. In the same vein, Wilde et al. (2014) genotyped rs16891982 (*SLC45A2*), rs12913832 (*HERC2*) and rs1042602 (*TYR*) in 63 Ukranian 5,000 year-old-samples (from the Eneolithic and Bronze Age) and estimated selection coefficients for these SNPs. They found evidences of positive selection acting on these genes favoring depigmentation of hair, skin and eyes after the time period represented by their population (6,500–4,000 years ago).

In our work, lack of diversity in the coding region of this locus resulted in a lack of power to detect selection. Thus, we decided to assess the existence of selective pressures on the immediately flanking intron 5, which is close to the SNP L374F and is large enough to contain enough variability for neutrality tests. Our observations were in agreement with those from Soejima et al. [25], as we found a strong evidence of positive selection flanking the exon containing the SNP L374F. Interestingly, the combined DHEW test, was only significant for South Europeans (TSI and IBS from 1KGP) after multiple tests correction. Similarly, the EHH test showed that the extension of reduced heterozygosity around allele 374F is significantly larger than expected under neutrality. This selective sweep is a strong signature of the selection acting on this allele in South Europeans. It seems likely that selection is favoring the 374F variant in Europe, as a mechanism of depigmentation. By resequencing intron 5 we also found one SNP (rs35397) that showed significant allele frequency differences between the least and most pigmented subsets of individuals in our sample. Functional assays for this mutation have not been performed yet, but our results suggest that it could be a cis-acting mutation that may regulate the expression of the gene and contribute to the effect of the SNP L374F. We also acknowledge, however, that this association could be due to the fact that it is in linkage disequilibrium with L374F, whose functional impact has already been described [37]. Further functional work should be done in order to ascertain the significance of this SNP in relation to pigmentation variability. Our estimates for the age of 374F placed the origin of this allele in Europe within the last 10,690–36,070 years (with a selection coefficient of 0.0243) using the individuals from the Hapmap, and within the last 25,270–35,290 years (with a selection coefficient of 0.0127) using the individuals from the 1KGP. These estimations support the work by Beleza et al. [42], who reported that the age of the allele ranged between 8,260–31,780 years under a dominant model and between 6,188–26,964 years under an additive model, after the arrival of modern humans in Europe, and similarly to other light pigmentation alleles such as allele A of rs1426654 in *SLC24A5*, allele G of rs2733831 in *TYRP1* [42]. Using the individuals from the 1KGP provides a lower selection coefficient and thus an older age of expansion. These differences could probably be due to the distinct genotyping coverage of both databases. While the HapMap data is subject to ascertainment bias of SNPs chosen for genotyping, the genomic data from the 1KGP can provide much deeper and informative genotype sequences, including alleles with a frequency lower than 5%. In this regard, it is also likely that the data from the 1KGP can also include some false positives due to the inherent errors derived from next-generation sequencing technology. Anyway, these estimations, regardless of the data set used, clearly place the date for the onset of selective sweep of allele 374F in Europeans in a period after the Out of Africa event.

The adaptive value for depigmentation is consistent with the vitamin D hypothesis proposed by Loomis [43] who suggested that depigmented skins would allow for the correct synthesis of vitamin-D, which requires that UV light penetrates into the skin. This, however, seems to cause collateral damage by increasing the susceptibility to melanoma. Melanoma is a tumor that develops from melanocytes, and its population incidence is generally associated with constitutive skin color and the geographic area of residence. Thus, the areas with a higher melanoma risk are Australia and New Zeland, where the intensity of UV radiation is very high and a great proportion of the population is light skinned [44]. In North America, for instance, the incidence rates of malignant melanoma in light skinned individuals have been shown to be five times higher than in Hispanics, and twenty times higher than in African Americans [45]. Our results clearly supported that the 374L variant, which was associated with darker skin, hair and eye color, is protective for melanoma, in agreement with previous studies [27],[28],[29], while the 374F, which is nearly fixed in light skinned European populations, leads to increased melanoma susceptibility.

A conciliatory explanation is that, as melanoma is commonly a late onset disease that normally develops after the reproductive age, its effect on the fitness of the individuals is not expected to be substantial, and thus, its prevalence is likely not to be shaped strongly by natural selection. However, the melanoma incidence in young people in the reproductive age has been reported to be increasing in some European countries. In particular, the increasing rate per year in young Spanish is around 8% [46]. However, despite the increasing incidence rates of melanoma, a decreasing mortality rate has also been recently described, probably due to improved early diagnosis [47], thereby not likely affecting the reproductive success of the individuals. This contrasting effect in melanoma susceptibility derived from the direct effect of natural selection has recently been observed also in the evolution of the pigmentary gene *MC1R* in Europeans [30].

In conclusion, we propose that natural selection in South Europeans is favoring the allele 374F. It might be involved, together with other genes like *MC1R* and others, in the process of depigmentation of European populations. The benefit of depigmentation in low UV radiation regions would outweigh the negative consequences of increased susceptibility to melanoma, which by being mainly a late-onset disease is not expected to affect the fitness of the individuals.

## Materials and Methods

### Ethics statement

This study was approved by the Ethics Committee of Cruces and Basurto University Hospitals (Bizkaia, Spain). Written informed consent was obtained from all subjects. The melanoma sample collection is recorded in the Spanish Carlos III Heath Institute (number: C.0002121).

### Phenotype characterization

We measured the constitutive pigmentation to 500 unrelated Spanish adult individuals living in the Basque Country by reflectance spectrometry. The collection of samples was performed during the winter in order to minimize the putative effects of solar exposure. Measures were taken on the inner surface of the upper arm, approximately mid-way between the axila and the medial epicondyle of the humerus, using a reflectance spectrophotometer EEL DS29 Digital Unigalvo with filter 609, which measures at 685 nm and is optimal for melanin quantification [2]. From each subject, a saliva sample was collected and the following variables were recorded: sex, age, eye and hair color, and skin phototype according to the Fitzpatrick scale [48]. DNA was then isolated from the saliva samples following a standard phenol-chloroform method.

### Population stratification analysis

To explore the presence of population stratification in the 34 most and the 34 least pigmented individuals from the 500 Basque samples collected, these individuals were genotyped using the Genome-wide Human SNP Array 6.0 (Affymetrix, Santa Clara, CA) in the facilities of the Centro Nacional de Genotipado (CEGEN) in Santiago de Compostela (Spain). Data management was performed with PLINK ver. 1.07 (http://pngu.mgh.harvard.edu/purcell/plink) [49]. Sample relatedness was accounted for by considering the PI_HAT value, which was set to be <0.2. We removed those SNPs with genotyping rates <97%, as well as those in chromosomes X, Y and mtDNA. We also excluded those SNPs that were not in Hardy-Weinberg equilibrium (conservatively set at p<0.05) and those in linkage disequilibrium based on pairwise genotypic correlation (window size = 50; step = 5; pairwise threshold = 0.2). After applying all the filters, a total of 106,521 SNPs were considered for subsequent analysis. We calculated p values for each SNP with PLINK and by means of a script written in R we generated a quantile-quantile (Q-Q) plot to assess the deviation from a theoretical χ^2^ distribution. Population stratification was assessed by means of EIGENSOFT 4.2 package (http://genetics.med.harvard.edu/reich/Reich_Lab/Software.html), which computes the Tracy-Widom statistics to evaluate the statistical significance of each principal component identified [50].

To further assess a possible substructure in the population under study, we typed 15 STRs (Identifiler Kit; Life Technologies, Carlsbad, CA) in the above samples. Amplified products were analyzed using an ABI 3500 Genetic Analyzer (Life Technologies) and allele designations were made with the aid of allelic ladders provided by the manufacturers. Statistical evaluations were performed using GDA [51] and PowerStats (http://www.promega.com/geneticidtools) software packages. Results were analyzed with the software STRUCTURE (v.2.3.3), which uses a Bayesian model-based clustering algorithm to infer the number of genetically distinct groups represented in a sample [52],[53]. We used 10 iterations to obtain the run with the highest penalized log-likelihood score based on the number of assumed populations (k), from k = 1 to k = 4, using correlated allele frequencies and an admixture model which enables a greater power to detect similar but distinct populations [54]. Each run consisted of a burn-in of 10,000 MCMC (Markov chain Monte Carlo method) steps, followed by 20,000 replications to estimate the allele frequencies in each of the k populations and, for each individual, the proportion of its genome derived from each population. We also resampled different subsets of SNPs (200, 500 and 1,000) genotyped with the Genome-wide Human SNP Array 6.0 (Affymetrix). Each random subset was created by choosing loci without replacement. In all cases, the data were shuffled 2,000 times. Linkage disequilibrium was tested using shuffling test for all possible combinations between loci obtaining an exact probability higher than 0.05 indicating independence of loci in all cases.

We also investigated the population structure by estimating the individual ancestry using the ADMIXTURE software [55]. The samples included for this analysis were the following: 223 East Africans, 190 West Africans, 125 North Africans, 3 South Africans, 168 from Near East and 210 from Western Europe [56],[57],[58],[59],[60] (Table S1). We explored k = 2–10 ancestral populations and performed 10 iterations with random seeds for each k value. We also calculated cross-validation errors for every run. Furthermore, standard errors for k = 6 and k = 7 were estimated using 200 bootstrappings by resampling subsets of each chromosome.

### DNA resequencing

Out of the 500 individuals, we resequenced the coding region and intron 5 of *SLC45A2* in the 34 most and 34 least pigmented individuals according to the reflectance distribution. The primers for the initial amplification of the coding region of *SLC45A2* from genomic DNA were designed by means of VariantSEQr Resequencing System software (Life Technologies). The obtained primer sequences were extended with a short M13 sequence to allow direct resequencing of the amplified products using universal M13 primers. Primers for the amplification and resequencing of intron 5 were designed with Primer3 (http://primer3.sourceforge.net). Primer sequences (5′->3′) for both the coding and the intronic regions are shown in Table S5.

Initial amplification of the coding region DNA was performed with the following cycling conditions: 1 cycle of 5 minutes at 96°C; 40 cycles of 30 seconds at 94°C, 45 seconds at 60°C and 45 seconds at 72°C; and a final step of 10 minutes at 72°C. Cycling conditions for the amplification of the intron 5 were: 5 minutes at 96°C, 40 cycles of 30 seconds at 94°C, 45 seconds at an optimized annealing time for each pair of primers, and 45 seconds at 72°C, and ending with a final step of 10 minutes at 72°C.

PCR products were purified with Excela Pure PCR Purification System (Edge BioSystems, Gaithersburg, MD). Sequencing reactions were performed in a 3500 Genetic Analyzer with BigDye 3.1 (Life Technologies). Thermocycling conditions included a denaturation step at 96°C for 5 minutes, followed by 30 cycles of 94°C for 20 seconds, 56°C for 20 seconds and 60°C for 4 minutes, plus a final extension cycle of 60°C for 10 minutes. Sequencing cleanup was performed with Performa DTR System (Edge BioSystems). Sequences were manually edited with Genalys v2.8 (http://software.cng.fr) and haplotypes were estimated with fastPHASE (http://stephenslab.uchicago.edu/software.html#fastphase). Sequences have been deposited in GenBank with accession numbers KF860209-KF860344. Estimation of linkage disequilibrium (D’ and r^2^) was performed with Haploview (http://www.broadinstitute.org/scientific-community/science/programs/medical-and-population-genetics/haploview/haploview).

### Genotyping

Genotyping of L374F was performed with KASPar (LGC Genomics KBioscience) in a StepOne Real-Time PCR System (Life Technologies) using approximately 20 ng of each DNA sample. Thermocycling conditions were the following: denaturation at 94°C for 15 minutes, 10 cycles of 94°C for 20 seconds and 61°C for 60 seconds dropping - 0.6°C/per cycle until 55°C; then 26 cycles of 94°C for 10 seconds and a 55°C for 60 seconds, and final step at 25°C for 10 minutes, where fluorescence was measured.

### Association testing with eye and hair color: samples and statistical analysis

We genotyped SNP L374F in 344 individuals from the sample of 500 Spanish living in the Basque Country (those encompassing the central value of the distribution of skin reflectance, excluding the extremes) collected by us, for whom we had information about hair and eye colour. To detect the association of F314L variant to these traits, we used the Fisher's exact test implemented in R (library *allelic*) and the R-package *SNPassoc* (http://www.creal.cat/jrgonzalez/software.htm) [61]. Hardy-Weinberg equilibrium was calculated with the library *HardyWeinberg* for R (http://cran.r-project.org/web/packages).

### Geographical distribution

We also genotyped SNP L374F (rs16891982) in an independent sample of 528 unrelated Spanish individuals obtained from the Spanish National DNA Bank for whom we had available information about their province of origin up to two generations (http://www.bancoadn.org/en/home.htm), plus a sample of 30 individuals from the Canary Islands (a subset of those described in [62], selected based on the lower ancestry to Northern Africa).We plotted the allele 374F frequencies in a map of Spain by means of Quantum GIS v 1.8.0 (http://www.qgis.org/). For that, we divided the map of the Iberian Peninsula in five homogeneous regions according to the total annual intensity of UV irradiation (data provided by the Spanish National Meteorological Agency), ranging from 23,500 to 34,500 J/m^2^. We also included the Canary Islands with an annual average of UV irradiation of approximately 43,400 J/m^2^.

### Diversity and selection tests at *SLC45A2*

To assess selection acting at this locus, we downloaded genotypes for the whole *SLC45A2* region (intronic and exonic) of the European samples from the 1000 Genomes Project (1KGP) (Phase 1 data from May 2011) by means of SPSmart [63]. This data set consisted of the following samples: 268 North Europeans (which included 87 Utah residents with Northern and Western European Ancestry, 88 British in England and Scotland and 93 Finnish in Finland) and 112 South Europeans (which included 98 Toscani from Italy and 14 Iberian from Spain).

The following population genetic parameters for the Basque samples resequenced in this study and for the populations in 1KGP were estimated using DnaSP 5.10 [64]: Tajima's D, Fu and Li's D, Fay and Wu's H and Fay and Wu's H normalized (using the chimpanzee as the outgroup). We also performed the DHEW combined test [33] with software kindly provided by K. Zeng. This test combines the DH test (a compound of Tajima's D and Fay and Wu's H) with Ewens-Watterson (EW) test and it is more powerful in detecting positive selection, as it has been proved to be insensitive to background selection and demographic changes such as population bottleneck or subdivision [33]. The p values for Tajima's D, Fay and Wu's H, E test and DHEW test were assessed through coalescent simulations using msHOT software [65] under the demographic model proposed by [32].

### EHH test

Recent positive selection acting on the specific allele 314F (rs16891982) in European populations was also assessed with an *in-house* implementation of the EHH method [66]. Briefly, we downloaded the genotypes for 100 kb upstream and 100 kb downstream from rs16891982 for the samples available from South European population (IBS+TSI; n = 112 individuals) from the 1KGP. The average mutation rate (µ) of each region was calculated from sequence divergence from the chimpanzee (assuming a 6 million year divergence time and the Jukes and Cantor model [67]) (sequence downloaded from UCSC genome browser web page http://genome.ucsc.edu/). The mutation rate was used to estimate the diversity parameter θ and the recombination parameter ρ needed to perform the simulations. The recombination parameter ρ was obtained from the recombination rates provided elsewhere [68]. In each simulation θ and ρ values were randomly sampled from a normal distribution with a mean equal to the estimation and a standard deviation equal to the mean. Recombinational hotspots were taken into account for the simulations. The basal recombination rate was calculated as the average of all the recombination rates, excluding the hotspots. Then, we calculated the EHH values by means of a Perl script and compared our real data with simulations following a neutral evolution model using the parameters from the demographic models inferred by Gutenkunst et al. [32]. Simulations were run by means of msHOT software [65], embedded within a customized Perl script. Simulations were then filtered maintaining those satisfying that the number of SNPs in each set of simulated haplotypes was similar to that observed in the real data, and that the number of haplotypes with the core haplotype was equal to that observed. For each region, several tens of thousands simulations were needed to obtain a total of 502 simulations for the upstream region, and 543 for the downstream region that satisfied the criteria. The msHOT command lines utilized for this were:

a) For the downstream region of the SNP: ./msHOT 224 500 -t tbs -r tbs 100000 -v 5 12348 15952 4 15953 18157 27 18158 18718 3 36693 39560 5 64619 66167 5 -I 3 0 224 0 -n 1 1.68202 -n 2 3.73683 -n 3 7.29205 -g 2 116.010723 -g 3 160.246047 -ma x 0.881098 0.561966 0.881098 x 2.79746 0.561966 2.79746 x -ej 0.028985 3 2 -en 0.028985 2 0.287184 -ema 0.028985 3 x 7.29314 x 7.29314 x x x x x -ej 0.197963 2 1 -en 0.303501 1 1

b) For the upstream region of the SNP: ./msHOT 224 500 -t tbs -r tbs 100000 -v 3 71 5719 5 5720 8493 15 8494 9611 5 -I 3 0 224 0 -n 1 1.68202 -n 2 3.73683 -n 3 7.29205 -g 2 116.010723 -g 3 160.246047 -ma x 0.881098 0.561966 0.881098 x 2.79746 0.561966 2.79746 x -ej 0.028985 3 2 -en 0.028985 2 0.287184 -ema 0.028985 3 x 7.29314 x 7.29314 x x x x x -ej 0.197963 2 1 -en 0.303501 1 1.

After this, the EHH values at points distant from the first SNP at 0.1, 0.2, 0.3, 0.4 and 0.5 units, in a scale of 0 to 1 (1 being equivalent to 100 kb) in this case, were recorded and the individual simulations showing an EHH value at the 95^th^ percentile at each point were selected. All simulations selected by the above process were lumped together. Then, duplicated points were removed and we forced that all points satisfied the condition EHH_i+1_ ≤ EHH_i_, where *i* increases from SNP number 1 in any direction (upstream or downstream). The resulting distribution of EHH values was used as the 95% cut-off line in the EHH analysis. Graphical representation of EHH values was performed using the R libraries plyr [69], reshape2 [70] and RColorBrewer (http://colorbrewer.org).

### Tajima's D for specific haplogroups

As a further test for positive selection acting on *SLC45A2* specific alleles, we performed a Tajima's D test for specific haplogroups, as previously described [30]. In brief, we obtained the haplotype sequences of a 5 kb region downstream from SNP L314F for the samples available from South European population (IBS+TSI; n = 224 haplotypes) from the 1KGP. We chose to perform this test on the downstream region as it is where intron 5 locates and we had already found enough variability in that region to perform the test. We grouped those haplotypes according to their ancestral or derived state at L314F, and performed Tajima's D test for each so-defined specific haplogroup with DnaSP. To obtain the p-value we run coalescent simulations with msHOT following a neutral evolution model but considering the demographic model previously described [32]. In these simulations, we predefined a core SNP at the last position emulating the L314F SNP (0 = ancestral; 1 = derived) and we accepted only those simulations with frequencies of the alleles at the core SNP similar to those observed in real data (allowance of +/−10%). We then extracted the simulations with a) the derived state and b) the ancestral state at the core SNP, and retained only those simulations with the same number of segregating sites than the observed data (allowance +/− 20%). After applying all these filters, a total of 579 simulations for the derived state and 224 for the ancestral state were considered. We finally calculated the Tajima's D value for each simulation with *msstats* software (http://molpopgen.org/software/msstats). From the distribution of all the values obtained, we then inferred the p-value for the Tajima's D test specific for the haplogroups containing the derived (314F) or the ancestral (314L) allele.

### Estimation of the age of expansion of allele 314F

We obtained a maximum likelihood estimation of the selection coefficient and the age of selective sweep of allele 314F in Europe using 2 data sets: a) the SNP data covering a region of 600 kb for a subset of 82 unrelated CEU individuals obtained from the Phase I of the 1KGP. We downloaded the haplotypes, which were already phased using SHAPEIT2 [71], and b) the phased haplotypes of 60 CEU individuals covering a region of 700Kb downloaded from the HapMap server (http://www.hapmap.org). For both datasets, starting from the putatively selected mutant position (314F), we first ran a hidden Markov model to identify ancestral haplotypes around the vicinity of the putatively selected mutant. We recorded the end points of each ancestral haplotype and used them as input for the parameter inference. We implemented the method by Chen and Slatkin [72] to infer the parameters including the selection intensity s and the age of selective sweep of the allele (see the original paper for the details of the method).

### Association testing with melanoma risk: samples and statistical analysis

We also genotyped L374F in a further set of 119 melanoma samples from the Basque Country collected by us. To detect the association of L314F variant with melanoma, we used the Cochran-Armitage Trend Test implemented in the R package *MaXact.* Hardy-Weinberg equilibrium was assessed using the package *HardyWeinberg* for R (http://cran.r-project.org/web/packages).

## Supporting Information

Figure S1 **QQ-plot generated with the p values for each SNP.** The overlap between the expected and observed p values indicated lack of stratification, supported by the value of the inflation factor of 1.
(TIF)

Figure S2 **Standard error estimates of individual ancestries for ADMIXTURE.** On the x-axis results for each k population are represented. Cross validation errors are lowest at k = 6 and k = 7.
(TIF)

Figure S3 **Admixture map for ancestral populations (k) = 7.** Each vertical line represents an individual from the corresponding population. Different colors indicate the ancestry proportions. The samples inside the black square correspond to the samples analyzed in this work. D: most pigmented individuals from our samples, L: least pigmented individuals from our sample.
(TIF)

Table S1 **Sources and sample sizes used for ADMIXTURE.**
(DOCX)

Table S2 **Haplotype frequencies for the coding region and intron 5 of** ***SLC45A2*** **in the groups of the most and least pigmented individuals.**
(DOCX)

Table S3 **Genotypic frequencies for each category of hair and eye color.** Fisher's Exact test showed significant differences in the frequencies of each genotype among hair color phenotypes, but not among eye color phenotypes. The association of the variant with each hair/eye color category was assessed with SNPassoc under an additive model and a 95% confidence interval. The ancestral allele G (374L) was associated with black (OR = 2.14; p = 0.0018) and dark brown hair (OR = 2.24; p = 0.0189), and the darkest eye color (brown/black; OR = 1.89; p = 0.0082).
(DOCX)

Table S4 **Frequencies of the SNPs not significantly associated with pigmentation variability found in intron 5 of** ***SLC45A2*** **in the least and most pigmented individuals.**
(DOCX)

Table S5 **Primers used for the resequencing of the coding region and of intron 5 of** ***SLC45A2*** **.**
(DOCX)
