# Supplementary figures and images for: The Interplay between Natural Selection and Susceptibility to Melanoma on Allele 374F of SLC45A2 Gene in a South European Population
Source: PLoS One. 2014 Aug 5;9(8):e104367. doi: 10.1371/journal.pone.0104367 (PMC4122405; doi:10.1371/journal.pone.0104367)

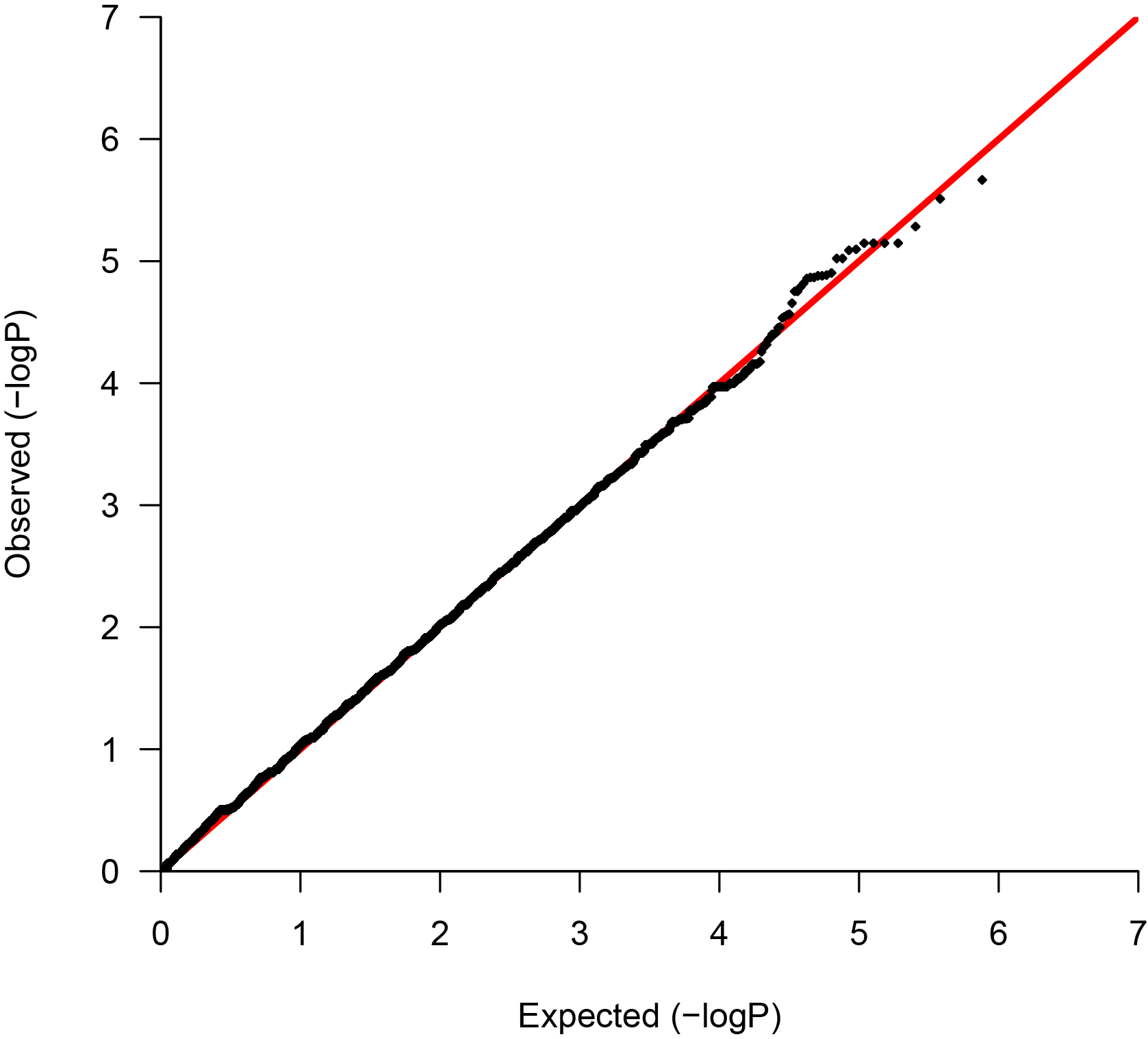

Supplement: Figure S1 — QQ-plot generated with the p values for each SNP. The overlap between the expected and observed p values indicated lack of stratification, supported by the value of the inflation factor of 1. (TIF) [file pone.0104367.s001.tif]

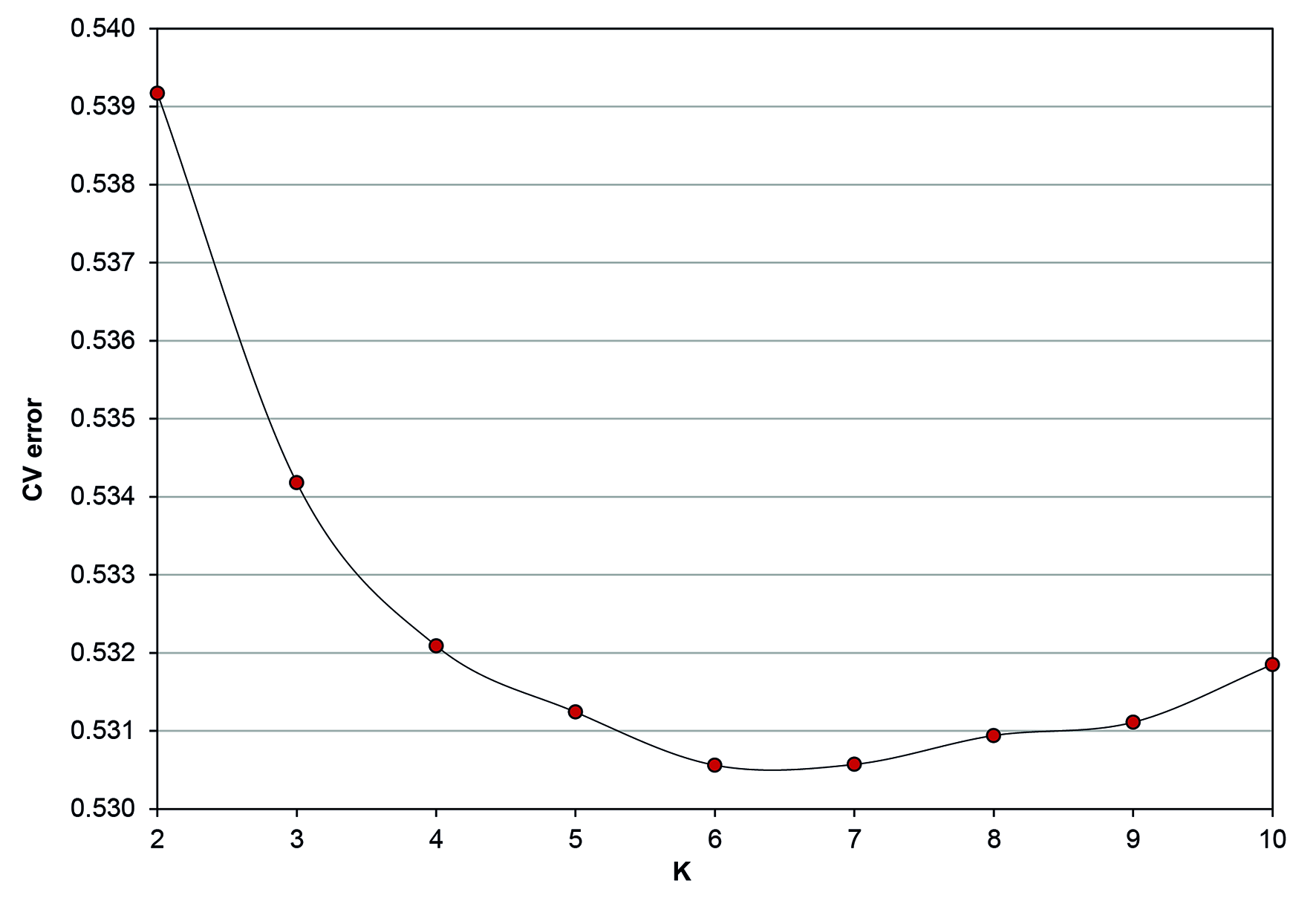

Supplement: Figure S2 — Standard error estimates of individual ancestries for ADMIXTURE. On the x-axis results for each k population are represented. Cross validation errors are lowest at k = 6 and k = 7. (TIF) [file pone.0104367.s002.tif]

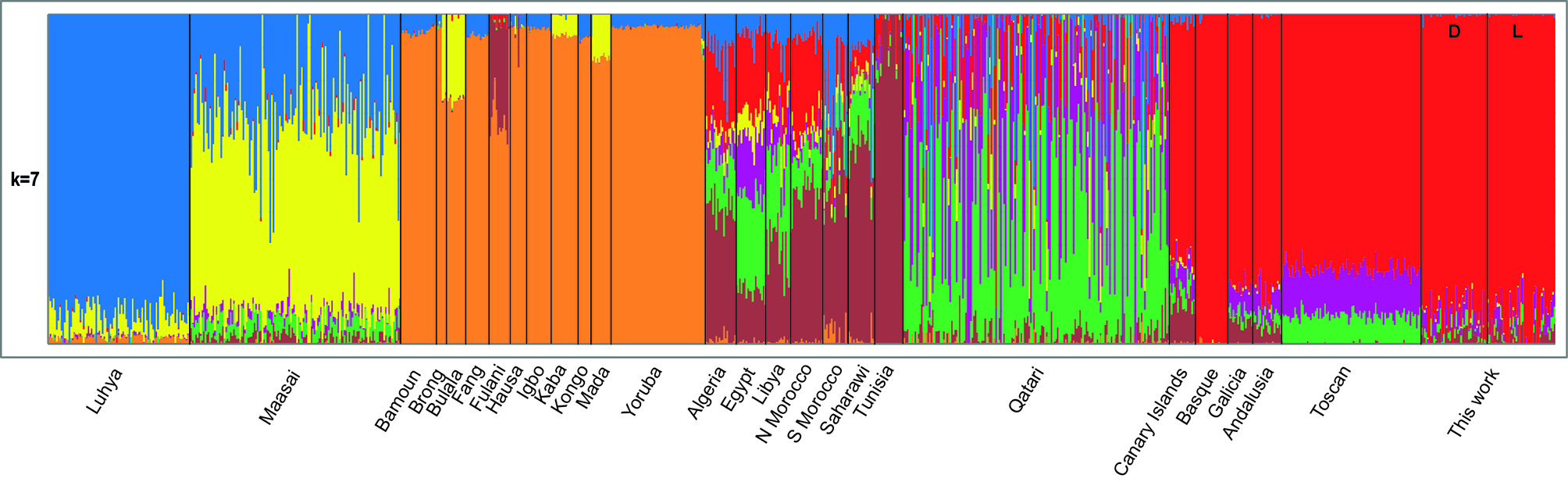

Supplement: Figure S3 — Admixture map for ancestral populations (k) = 7. Each vertical line represents an individual from the corresponding population. Different colors indicate the ancestry proportions. The samples inside the black square correspond to the samples analyzed in this work. D: most pigmented individuals from our samples, L: least pigmented individuals from our sample. (TIF) [file pone.0104367.s003.tif]
