# Supplementary material for: The Interplay between Natural Selection and Susceptibility to Melanoma on Allele 374F of SLC45A2 Gene in a South European Population
Source: PLoS One. 2014 Aug 5;9(8):e104367. doi: 10.1371/journal.pone.0104367 (PMC4122405; doi:10.1371/journal.pone.0104367)
Supplement: Table S1 — Sources and sample sizes used for ADMIXTURE. (DOCX) [file pone.0104367.s004.docx]

**Table S1.**

| **Region** | **Population** | **n** | **Reference** |
| --- | --- | --- | --- |
| East Africa | Luhya (Kenya) | 90 | The International HapMap 3 Consortium. [56] |
|  | Maasai (Kenya) | 133 | The International HapMap 3 Consortium. [56] |
| West Africa | Bamoun (Cameroon) | 18 | Bryc et al. [57] |
|  | Brong (Ghana) | 8 | Bryc et al. [57] |
|  | Bulala (Chad) | 15 | Bryc et al. [57] |
|  | Fang (Cameroon) | 15 | Bryc et al. [57] |
|  | Fulani (Nigeria) | 12 | Bryc et al. [57] |
|  | Hausa (Nigeria) | 12 | Bryc et al. [57] |
|  | Igbo (Nigeria) | 15 | Bryc et al. [57] |
|  | Kaba (Nigeria) | 17 | Bryc et al. [57] |
|  | Kongo (Congo) | 9 | Bryc et al. [57] |
|  | Mada (Nigeria) | 12 | Bryc et al. [57] |
|  | Yoruba (Nigeria) | 57 | Bryc et al. [57] |
| South Africa | Xhosa (South Africa) | 3 | Bryc et al. [57] |
| North Africa | Algeria | 19 | Henn et al. [58] |
|  | Egypt | 19 | Henn et al. [58] |
|  | Libya | 17 | Henn et al. [58] |
|  | Morocco_N | 18 | Henn et al. [58] |
|  | Morocco_S | 16 | Henn et al. [58] |
|  | Saharawi | 18 | Henn et al. [58] |
|  | Tunisia | 18 | Henn et al. [58] |
| Near East | Qatari (Qatar) | 168 | Hunter-Zinck et al. [59] |
| Western Europe | Canary Islands (Spain) | 17 | Botigué et al. [60] |
|  | Basque (Spain_N) | 20 | Henn et al. [58] |
|  | Galicia (Spain_NW) | 17 | Botigué et al. [60] |
|  | Andalusia (Spain_S) | 17 | Botigué et al. [60] |
|  | Toscan (Italy) | 88 | The International HapMap 3 Consortium. [56] |
|  | Spanish (this work) | 68 | This work |
