# Supplementary material for: The Interplay between Natural Selection and Susceptibility to Melanoma on Allele 374F of SLC45A2 Gene in a South European Population
Source: PLoS One. 2014 Aug 5;9(8):e104367. doi: 10.1371/journal.pone.0104367 (PMC4122405; doi:10.1371/journal.pone.0104367)
Supplement: Table S2 — Haplotype frequencies for the coding region and intron 5 of SLC45A2 in the groups of the most and least pigmented individuals. (DOCX) [file pone.0104367.s005.docx]

| **Coding region** |  | |  |  |  |  |  |  |  |  |  |  |
| --- | --- | --- | --- | --- | --- | --- | --- | --- | --- | --- | --- | --- |
| **Haplotype** | **Frequency** | |  |  |  |  |  |  |  |  |  |  |
|  | least pigmented (n=68) | most pigmented (n=68) | **E272K** | **T329T** | **L374F** |  |  |  |  |  |  |  |
| H1 | 63 | 48 | G | G | C |  |  |  |  |  |  |  |
| H2 | 2 | 14 | G | G | G |  |  |  |  |  |  |  |
| H3 | 1 | 1 | G | A | C |  |  |  |  |  |  |  |
| H4 | 2 | 4 | A | G | C |  |  |  |  |  |  |  |
| H5 | 0 | 1 | A | A | C |  |  |  |  |  |  |  |
|  |  |  |  |  |  |  |  |  |  |  |  |  |
| **Intron 5** |  |  |  |  |  |  |  |  |  |  |  |  |
| **Haplotype** | **Frequency** | |  |  |  |  |  |  |  |  |  |  |
|  | least pigmented (n=68) | most pigmented (n=68) | **rs250416** | **rs142167897** | **rs35394** | **rs35395** | **rs142639084** | **rs35396** | **rs10080040** | **rs40132** | **rs35397** | **rs115658239** |
| H1 | 64 | 50 | C | C | T | C | C | A | A | A | T | G |
| H2 | 1 | 0 | C | C | T | C | C | C | A | A | T | G |
| H3 | 1 | 0 | A | C | C | T | G | A | A | G | G | G |
| H4 | 1 | 0 | C | T | T | C | C | A | A | A | T | G |
| H5 | 1 | 8 | C | C | T | C | C | A | A | A | G | G |
| H6 | 0 | 1 | C | C | T | C | C | A | A | A | T | C |
| H7 | 0 | 1 | C | C | T | C | C | A | T | A | G | G |
| H8 | 0 | 1 | A | C | C | T | G | C | A | G | G | G |
| H9 | 0 | 4 | C | C | T | T | C | A | A | A | G | G |
| H10 | 0 | 2 | A | C | C | T | C | A | A | G | G | G |
| H11 | 0 | 1 | A | C | C | T | C | A | A | G | T | G |

**Table S2**.
