# Supplementary material for: The Interplay between Natural Selection and Susceptibility to Melanoma on Allele 374F of SLC45A2 Gene in a South European Population
Source: PLoS One. 2014 Aug 5;9(8):e104367. doi: 10.1371/journal.pone.0104367 (PMC4122405; doi:10.1371/journal.pone.0104367)
Supplement: Table S3 — Genotypic frequencies for each category of hair and eye color. Fisher's Exact test showed significant differences in the frequencies of each genotype among hair color phenotypes, but not among eye color phenotypes. The association of the variant with each hair/eye color category was assessed with SNPassoc under an additive model and a 95% confidence interval. The ancestral allele G (374L) was associated with black (OR = 2.14; p = 0.0018) and dark brown hair (OR = 2.24; p = 0.0189), and the darkest eye color (brown/black; OR = 1.89; p = 0.0082). (DOCX) [file pone.0104367.s006.docx]

**Table S3.**

|  |  |  |  |  | ***SNPassoc:* Additive model** | | | |
| --- | --- | --- | --- | --- | --- | --- | --- | --- |
| **Hair color** | **Genotype** | **Freq.** | **HW** | **Fisher** | **OR** | **lower** | **upper** | **p-value** |
| **Black** | 374F/374F | 48 | 1 | 0.0002* | 2.14 | 1.33 | 3.43 | **0.0018**** |
|  | 374F/374L | 36 |  |  |  |  |  |  |
|  | 374L/374L | 1 |  |  |  |  |  |  |
| **Dark brown** | 374F/374F | 17 | 1 |  | 2.24 | 1.17 | 4.29 | **0.0189**** |
|  | 374F/374L | 14 |  |  |  |  |  |  |
|  | 374L/374L | 1 |  |  |  |  |  |  |
| **Brown** | 374F/374F | 158 | 0.9291 |  | 0.49 | 0.32 | 0.77 | **0.0016**** |
|  | 374F/374L | 39 |  |  |  |  |  |  |
|  | 374L/374L | 3 |  |  |  |  |  |  |
| **Blonde** | 374F/374F | 22 | 0.7304 |  | 0.22 | 0.05 | 0.95 | 0.0861 |
|  | 374F/374L | 2 |  |  |  |  |  |  |
|  | 374L/374L | 0 |  |  |  |  |  |  |
| **Red** | 374F/374F | 2 | 0.0818 |  | 1.17 | 0.13 | 10.85 | 1 |
|  | 374F/374L | 1 |  |  |  |  |  |  |
|  | 374L/374L | 0 |  |  |  |  |  |  |
|  |  |  |  |  | ***SNPassoc:* Additive model** | | | |
| **Eye color** | **Genotype** | **Freq.** | **HW** | **Fisher** | **OR** | **lower** | **upper** | **p-value** |
| **Brown/Black** | 374F/374F | 139 | 1 | 0.2023 | 1.94 | 1.2 | 3.14 | **0.0054**** |
|  | 374F/374L | 66 |  |  |  |  |  |  |
|  | 374L/374L | 4 |  |  |  |  |  |  |
| **Hazel** | 374F/374F | 43 | 1 |  | 0.72 | 0.38 | 1.37 | 0.3068 |
|  | 374F/374L | 11 |  |  |  |  |  |  |
|  | 374L/374L | 1 |  |  |  |  |  |  |
| **Green** | 374F/374F | 42 | 1 |  | 0.67 | 0.34 | 1.29 | 0.5109 |
|  | 374F/374L | 12 |  |  |  |  |  |  |
|  | 374L/374L | 0 |  |  |  |  |  |  |
| **Blue** | 374F/374F | 22 | 1 |  | 0.22 | 0.05 | 0.94 | 0.0863 |
|  | 374F/374L | 2 |  |  |  |  |  |  |
|  | 374L/374L | 0 |  |  |  |  |  |  |
| **Grey** | 374F/374F | 1 | 0.1893 |  | 2.03 | 0.19 | 22.15 | 0.4850 |
|  | 374F/374L | 1 |  |  |  |  |  |  |
|  | 374L/374L | 0 |  |  |  |  |  |  |

**** significant after Bonferroni correction**

**Freq**.=Absolut frequency of each genotype; HW=Hardy Weinberg equilibrium (p-value); **Fisher**=Fisher’s Exact Test (p-value); **OR**=Odd’s Ratio; **lower and upper** = confidence intervals.
