# Supplementary material for: The Interplay between Natural Selection and Susceptibility to Melanoma on Allele 374F of SLC45A2 Gene in a South European Population
Source: PLoS One. 2014 Aug 5;9(8):e104367. doi: 10.1371/journal.pone.0104367 (PMC4122405; doi:10.1371/journal.pone.0104367)
Supplement: Table S4 — Frequencies of the SNPs not significantly associated with pigmentation variability found in intron 5 of SLC45A2 in the least and most pigmented individuals. (DOCX) [file pone.0104367.s007.docx]

**Table S4.**

| **SNP** | **Change** | **Frequency** |  |
| --- | --- | --- | --- |
|  |  | Least pigmented (n=68) | Most pigmented (n=68) |
| rs250416 | C>A | 0.015 | 0.059 |
| rs142167897 | C>T | 0.015 | 0 |
| rs35394 | T>C | 0.015 | 0.059 |
| rs35395 | C>T | 0.015 | 0.102 |
| rs142639084 | C>G | 0.015 | 0.015 |
| rs35396 | A>C | 0.015 | 0.015 |
| rs10080040 | A>T | 0 | 0.015 |
| rs40132 | A>G | 0.015 | 0.059 |
| rs115658239 | G>C | 0 | 0.015 |

n=chromosomes
