# Supplementary material for: The Interplay between Natural Selection and Susceptibility to Melanoma on Allele 374F of SLC45A2 Gene in a South European Population
Source: PLoS One. 2014 Aug 5;9(8):e104367. doi: 10.1371/journal.pone.0104367 (PMC4122405; doi:10.1371/journal.pone.0104367)
Supplement: Table S5 — Primers used for the resequencing of the coding region and of intron 5 of SLC45A2 . (DOCX) [file pone.0104367.s008.docx]

**Table S5.**

| Region | Primer sequences (5’- 3’) | Amplicon size |
| --- | --- | --- |
| Exon 1 | 1FW TGTAAAACGACGGCCAGTCCACGAGCATCATGACTCCCA  1REV CAGGAAACAGCTATGACCCAACGTCTTATGGAGCCAGAGGG 1bFW TGTAAAACGACGGCCAGTCCTGTCTGCCCACCTTGTGC  1bREV CAGGAAACAGCTATGACCGGCCAGGCTCCACGTCAAAT | 591  508 |
| Exon 2 | 2FW TGTAAAACGACGGCCAGTAGCAGCCCATCAGCTGACCC  2REV CAGGAAACAGCTATGACCGCACCAGCCCTAAGCAACCA | 533 |
| Exon 3 | 3FW TGTAAAACGACGGCCAGTTTCCAAGGGATGATAGCTACGGG  3REV CAGGAAACAGCTATGACCTTGCCTCTGCTGTCTTCAGGG | 590 |
| Exon 4 | 4FW TGTAAAACGACGGCCAGTTGGCAGGCTCCGTAGTGTCC  4REV CAGGAAACAGCTATGACCTCTGGGCCTGAAACTGCCAA | 554 |
| Exon 5 | 5FW TGTAAAACGACGGCCAGTCGAACCTGCTGGGACTCATCC  5REV CAGGAAACAGCTATGACCTGCGACTTGGTCATGACAGCAT | 586 |
| Exon 6 | 6FW TGTAAAACGACGGCCAGTGGTTGCAGTTGGTTGGGCAT  6REV CAGGAAACAGCTATGACCCCAAGGCAATTTCAAGCTGTCC | 568 |
| Exon 7 | 7FW TGTAAAACGACGGCCAGTAGCCTATCAGTGCCACCGCA  7REV CAGGAAACAGCTATGACCTGGAAAGCCAACTCAGCTCCA  7bFW TGTAAAACGACGGCCAGTGGCAGATTCCACGGCTGAAA  7bREV CAGGAAACAGCTATGACCGGTGGTTCTGTGCTTCCTTTGC | 382  383 |
| 5’UTR | UTRFW TGTAAAACGACGGCCAGTGTCACACATGGCTTCTCTCACGTT  UTRREV CAGGAAACAGCTATGACCAAGGCTTTATTACCTCACGGCCA | 581 |
| Intron 5 | INT1F TGCTCTTGGAATCAGTGGGT  INT1R GGCTGCATTGTACTTGCGAC  INT2F GCCAGCTTTTCTGAGCCTCT  INT2R TTCAAGGGCTGAGGGGACTA  INT3F TCTTCCTCTTCGAGTGTGCAA  INT3R AACCACCCTCAATGACCCAC  INT4F AATACCAGCCAGCTCTGCTC  INT4R TTTGCCTTCTCCTCTGCTGG  INT5F ATCAGACCCATCAGAGGGCA  INT5R GAGTTAGGGAGCCAGCGATG  INT6F CAGCTCAGCCTGTCTTGTTG  INT6R GTCTACTCCACCCTGGTCCT | 446  812  886  822  978  944 |
